# Beyond the taboo: a thanatosociological investigation of companion animal loss based on an interview study with small animal veterinarians

**DOI:** 10.3389/fvets.2026.1719122

**Published:** 2026-01-30

**Authors:** Svenja Springer, Christian Dürnberger

**Affiliations:** Department of Interdisciplinary Life Sciences, Messerli Research Institute, University of Veterinary Medicine Vienna, Vienna, Austria

**Keywords:** animal death, end-of-life care, grief, interview study, palliative medicine, small animals, veterinary medical ethics

## Abstract

Thanatosociology examines the social dimensions of death, focusing on how societies perceive and navigate the realities of mortality. A key argument in this field posits that, in certain contemporary Western societies, direct experiences with dying and death are increasingly rare, leading to the emergence of death as a taboo topic. Given the emotional bond between pet owners and their pets, it becomes essential to examine the implications of companion animal loss within this framework. As pets often die at home, this topic warrants investigation to better understand how these experiences can influence societal perceptions of mortality. This study aims to highlight this often-overlooked perspective, emphasizing the significance of companion animal loss in shaping collective attitudes toward death and dying. To investigate this phenomenon, qualitative interviews were conducted with 20 small animal veterinarians specializing in hospice and palliative care. The findings corroborate the notion of a taboo surrounding death, as veterinarians highlighted the physical and emotional distancing from dying individuals and the pervasive fear and uncertainty when confronting mortality. Further, respondents argued that other cultures engage with death more openly and suggest that the experience of accompanying a dying pet can challenge societal repression, thereby fostering a more reflective approach to death for both adults and children. Relatedly, veterinarians stated that this experience provides a unique opportunity to bring death into the public consciousness, transforming it from a taboo into a more visible aspect of life. Further, respondents described the loss of a companion animal as a “training ground” for confronting death, noting that past negative experiences can complicate decision-making regarding animal care. This research suggests that veterinarians are not only perceived as medical professionals but also as “experts on death,” providing critical emotional support during end-of-life care. The findings highlight the need to integrate discussions about death into veterinary education, emphasizing the critical role veterinarians play in facilitating conversations about mortality and supporting bereaved companion animal owners. By recognizing this dual role, we can enhance the capacity of the veterinary profession to address the complexities of grief and mortality, ultimately fostering a more open dialogue about these important issues.

## 1 Introduction

Thanatosociology deals with the social dimension of death and examines how societies perceive and cope with death. A widespread argument suggests that death and dying are more invisible in contemporary Western society than ever before. Foucault notes, for example, that the public rituals accompanying death are increasingly disappearing (1). Thus, death is no longer a grand ceremony in which the community participates, but something that is hidden; it has become a private, often even shameful matter. Similar observations can be found in the works of Ariès (2, 3) and Elias (4), who describe this change as a form of repression or taboo, where modern society ignores death out of discomfort or defense. However, in recent times, findings also point to opposing dynamics, such as the establishment of thanatosociology as a distinct discipline or civil society movements advocating for a more open approach to death and dying. Macho and Marek refer to this context as a “new visibility of death” (5). Nassehi argues that the significant discussions about death indicate that it is inaccurate to claim that society represses the topic of death (6). In other words, there are ambivalences between the tabooing or displacement of death and tendencies toward destigmatization or explicit discussion (7).

The fact that “death” and “dying” are experiencing a new surge of attention in media and academic discourses should not and cannot obscure the historical comparison that shows a decline in primary experiences with the dying and the dead (8–10). There are clear examples to illustrate this: Firstly, since the late 19th century, dying has spatially increasingly shifted to hospitals and nursing homes (11). While death was commonly experienced in the home environment in the past, it now predominantly occurs in institutions specifically established for this purpose. Data from Austria, for example, indicate that in 2019, out of over 82,000 deceased adults, nearly half (49%) died in hospitals, 20% in nursing homes, and only a quarter at home—and even among this 25%, it is questionable to what extent dying took place in a social environment (12). Secondly, this segregation is accompanied by professionalization and medicalization: it is no longer close relatives who accompany a person until death, but caregivers who take on this task as part of their profession. Concurrently, the dying process is increasingly discussed not as an existential phenomenon but more and more in the language of medicine (11). Particularly when dying is “outsourced” to specific institutions, the professionals working in these settings become “experts of death” (7), interpreting death in their own technical language “for us” (7). Thirdly, since primarily very old or seriously ill individuals die in the Global North, death is increasingly perceived as a problem of a specific subset of society, leading to a particularization of death (7). Fourthly, as Foucault already diagnosed, funerals and death cults are losing significance in modern society. Mourning is increasingly privatized and internalized; if it exceeds societal norms, it is viewed as pathological. Traditional forms of giving meaning to death are increasingly being displaced (13).

The arguments and examples presented convincingly support the argument of a shift and a decline in primary experiences with “death” and “dying”—however, this argument requires an important addition. It focuses on the death of *humans* while neglecting that other beloved companions also die within the fabric of society. More specifically, this article argues that death and dying are “back” in the immediate personal experience of many individuals, particularly through the death of their companion *animals*.

In 2024, 44% of German households owned a companion animal; among families with children, this figure was even higher at 68% (14). Most people view their animals as close friends or even family members (15). A recent study shows that 75% of over 2,000 surveyed cat and dog owners chose their animals clearly and explicitly as “companions” for themselves or their families (15). Human-animal relationships, in this context, are therefore generally characterized by deep emotional bonds, and for this reason, the loss of a companion animal—whether through natural death or euthanasia—can trigger a profound crisis for companion animal owners (16).

Even in a time when immense progress has been made not only in human medicine but also in veterinary medicine, one thing remains certain: the farewell to companion animals such as dogs and cats is inevitable. Despite the ability to treat diseases once considered incurable, a goodbye must come one day. When a companion animal approaches death, it usually happens in a familiar environment, making the accompanying process—a stark contrast to the “outsourced” dying of human beings—a personal and immediate experience. This intimate setting provides the opportunity, and indeed challenges, to confront the topics of dying and death directly, without the barriers, but also the “shields” that are often associated with the death of humans. This is exemplified in the mourning process of children and adolescents: A study with 32 children aged 5 to 18 identified that this group continues to maintain bonds with their deceased companion animals in order to process the loss (17). Given the widespread presence of companion animals, the intense emotional bonds between humans and animals, their dying process—which often occurs in the home environment—and their generally much shorter life expectancy compared to humans, it is evident that many children and young adults experience their first encounters with death and dying through their companion animals (16).

In the context of thanatosociology, it can be argued that the death of companion animals contributes to a “new visibility” of dying in our society. Against this backdrop, it seems appropriate to include the perspective of the professional group that operates at the intersection of humans, animals, and the dying process: veterinarians specializing in hospice and palliative care. Or, as Walter (7) puts it, the “experts of companion animal death.” In their daily work, they accompany both the animals and their owners and family members through the often emotionally taxing process of farewell (18–20).

Herewith, the veterinarian not only acts as a medical professional but also as an emotional support for the grieving individuals. Particularly by conducting house visits and accompanying the dying process in a personal environment, veterinarians gain insights into the dynamics of (familial) interactions with death that remain closed off to other professional groups (18–20). Their observations and experiences provide valuable insights into how experiencing the death of a companion animal can help dismantle societal taboos surrounding death and promote a more open engagement with this sensitive topic. Hence, through their professional proximity to everyday experiences of dying within the context of human-animal relationships, veterinarians possess specific experiential and interpretative knowledge that is of particular interest for the sociological analysis of contemporary death cultures.

Against this background, the present article presents the results of an interview study with veterinarians focusing on hospice and palliative care in small animal medicine. The overarching goal of the study is to explore, through qualitative interviews, how veterinarians engaged in palliative medicine and end-of-life care perceive societal discussions about dying and death, and what significance they attribute to the dying and death of companion animals in this context.

The two research questions are thus: How do veterinarians perceive societal discussions about dying and death? And, what significance do they attribute to the dying and death of companion animals in this context? The study argues that companion animals can contribute to facilitating primary experiences with dying and death, bringing these existential themes into a new societal visibility. It is expected that the results of the study will not only deepen the understanding of the human-animal relationship but also open new perspectives for dealing with death and mourning in contemporary society.

## 2 Materials and methods

This study is part of a larger project that not only addresses the research questions presented here but also explores significant aspects of hospice and palliative care in veterinary medicine (18), as well as work-life boundary management and coping strategies among veterinarians (21). The research is based on in-depth interviews (22) with 20 small animal veterinarians from Germany (*n* = 8), Switzerland (*n* = 7), and Austria (*n* = 5), all of whom have specialized in hospice and palliative medicine (certified by the International Association for Animal Hospice and Palliative Care, IAAHPC) and/or have prominently featured this specialization on their practice websites. Among the 20 interviewees, 19 were self-employed, while 1 was employed after many years as a self-employed professional. The study group comprised 18 women and 2 men. Detailed information regarding country of practice, gender, employment status (self-employed or employed), years of professional experience, interview duration, and interviewers is provided in the overview table in Supplementary material 1.

### 2.1 Recruitment process and study participants

To initiate the recruitment process for veterinarians participating in the study, an online search was conducted for veterinarians, practices, and/or clinics in the field of small animal medicine, targeting specific regions. For the Austrian participants, the platform Herold.at was used on June 16, 2022. The focus then shifted to German participants, with the search conducted via 11880.com from June 26 to 29, 2022. Finally, for Swiss participants, local.ch was utilized, with the search taking place on July 14, 2022. Additionally, to ensure a comprehensive reach, another online search was conducted using Google.at on August 6, 2022. Relevant search terms such as “veterinarian,” “palliative,” “hospice,” and “small animal practice” were used to identify 37 additional veterinarians, practices, and/or clinics in the small animal sector (Germany *n* = 28; Switzerland *n* = 2; Austria *n* = 7) that were not listed in the industry directories. These online searches aimed to assemble a diverse and qualified pool of veterinarians for the study. A total of 3,007 websites were reviewed. The results of the online search showed that only 52 websites (2.4%) mentioned the services of hospice and/or palliative care. From this 2.4%, potential study participants were selected based on various considerations, including diversity in practice type and specialization, professional experience, degree of urbanization, and workplace, to obtain a wide range of experiences and thus increase the likelihood of transferability. Overall, there were three recruitment phases. The first recruitment phase began on December 7, 2022, with outreach to nine selected veterinarians in Germany, five in Switzerland, and six in Austria. Following this initial phase, 16 veterinarians (seven in Germany, four in Switzerland, and five in Austria) confirmed their participation. In the second recruitment phase, on February 14, 2023, three German veterinarians and one veterinarian from Switzerland were contacted. During this phase, an additional veterinarian in Switzerland agreed to participate. In the third phase, on March 10, 2023, two veterinarians in Switzerland were contacted, with one veterinarian confirming participation on March 17, 2023, thereby completing the recruitment process.

In January 2023, two pilot interviews were conducted with veterinarians specializing in palliative and hospice medicine, aligning with the target population. The main objective of these pilot interviews was to assess the clarity and comprehensibility of the interview questions and to ensure that no important topics were overlooked. Based on feedback, it was confirmed that the questions were well-understood, and minor adjustments were made to improve the wording where necessary. A formal validation process (e.g., external expert evaluation or structured bias assessment) was not conducted. Since one of the two pilot interviews went smoothly, it was decided to include the corresponding data from this interview in the final sample of the study. Participants were offered a compensation of € 50 for their participation in the interview study. The study was submitted for review to the chair of the Ethics Committee of the Medical University of Vienna. After reviewing the study, the requirement for a commission-based review was waived.

During the recruitment process, it was challenging to find male veterinarians, as there are more women than men in this field. Therefore, it was decided to accept the low number of male participants in this sample. Before the study, all participants were informed about the nature of the data to be collected and how it would be handled. It was emphasized that participation was voluntary and that they could withdraw their consent at any time during or after the study. All participants provided written consent, and there were no withdrawals during or after the study.

### 2.2 Data collection and structure of interviews

All 20 interviews were conducted in German and online using a web conferencing tool (Cisco Webex), which also allowed for video recording. The duration of the interviews varied between 48 and 100 min, with an average length of 73 min. All interviews followed a semi-structured interview guide that consisted of four parts and six topics (Supplementary material 2).

The development of the interview guide was based on the theoretical framework proposed by Springer and Flammer (23). This framework includes four key aspects that are particularly relevant to veterinary hospice and palliative care: relationships, time, communication, and infrastructure. Additionally, the final fourth part addressed the topic of dying and death of animals and its influence on contemporary society. The following three questions were designed to elicit responses and reflections on this topic:

- Has your perspective on dying and death changed through your work in palliative care and/or end-of-life support in general? If so, how?
- Do you believe that companion animal owners who consciously choose palliative care and end-of-life support for their animals have a different perspective on dying and death, or do they develop one during the process?
- In your opinion, what influence do the dying and death of animals have on our approach to dying and death in society at large?

Individual questions were slightly rephrased during the interviews and tailored to each participant and their professional background. The first author of this article (S.S.) conducted 18 interviews, while the last author (C.D.) conducted two interviews.

### 2.3 Data analysis

The recordings were transcribed verbatim and coded using the software program MAXQDA 2020 (Version 20.4.2; Berlin, Germany). Following the template-organizing style (24), categories and codes were developed and formulated based on the key aspects of the interview guide, the research questions, and the project's hypotheses. A first and second cycle of coding was conducted (25) to analyze the data obtained from the 20 interviews. Using a deductive approach, the overarching goal of the first coding cycle was to summarize data sections and categorize similar data units (25).

The deductive approach in the first coding cycle was guided by the conceptual framework introduced by Springer and Flammer (23), which identifies key areas in veterinary hospice care. These areas provided the initial categories for coding and ensured alignment with the pre-established theoretical framework. The original coding list for the first coding cycle included nine categories with a total of 40 codes. During the first coding cycle of two interviews (conducted by S.S. and C.D.), this original list was adjusted to include two new codes and to modify existing codes for better applicability in the analyses (25). These changes primarily followed an inductive approach based on the data obtained. The final coding list includes nine categories with 42 codes (Supplementary material 3).

Categories and, in particular, codes were continuously discussed within the project team (S.S. and C.D.) to ensure the relevance and application of the codes, especially during the first coding cycle. In the second coding cycle, the initial results were grouped into smaller categories and clusters to obtain more meaningful units for the subsequent content analysis. The interviews were conducted and transcribed in German, the categorization and coding framework was ultimately developed in English. During this translation process, attention was paid to potential conceptual and cultural differences that might arise. To mitigate possible issues, a collaborative approach was chosen, where both authors engaged in discussions about the nuances of language and meaning. Although minor linguistic challenges arose with certain quotes, care was taken to ensure that their essence was preserved by selecting translations that accurately reflected the original context. Furthermore, specific cultural constructs inherent in the German language that may not have direct English equivalents were acknowledged.

This qualitative interview study is exploratory in nature, aimed not at making statistical generalizations about a broader population, but rather at examining the arguments surrounding displacement theses within this specific context. The research seeks to gain insights into how the loss of companion animals may influence perceptions of dying and death, including the potential for tabooization in our society.

## 3 Results

### 3.1 Dying and death in Western society

#### 3.1.1 Taboo topic death and cultural differences

The results of the study show that the topics of dying and death—including the possibility of end-of-life care—are treated as a taboo from the perspective of the respondents. A veterinarian states that “*accompanying until death is somewhat taboo, even in the field of human medicine”* [Interview 6, Position (Pos.) 15]. Another veterinarian notes, “*Death is part of life. That's something I see; it is being anonymized; it is simply not discussed”* (Interview 16, Pos. 32). In this context, several interviews referenced experiences and narratives from other cultures where aging, dying, and death are perceived and lived differently according to the respondents. A veterinarian recounts that one of her employees comes from a Southeastern European country, “*where they have a completely different approach to death, because they prepare the deceased and sing lamentations, and have, for example, a very different relationship with death. […] I don't believe we will ever have this access in Austria or in our world here […]. […] There is a different approach to it.”* (Interview 13, Pos. 151).

Another veterinarian emphasizes this with her experiences abroad: “*I spent some time in India, and I find that they handle these topics very differently. They are simply part of life there, and I am sometimes appalled by how […] this is completely new territory for them [people in Western society], and they often do not want to deal with it at all […]”* (Interview 14, Pos. 152). In another interview, it is stated similarly: “*This is certainly a topic that is treated very negligently in Austria, that everything unpleasant, moving towards dying and death, is not discussed.”* (Interview 10, Pos. 103).

A veterinarian compares the different rituals of memorial cultures around “All saints' day” at the beginning of November and notes: “*Look at Mexico or South America, the D*í*a de Muertos, where the deceased relatives are remembered. There are flowers, there are dishes, people go to the cemetery, they cook something nice for them. That may seem silly to us, but it is a wonderful way of dealing with deceased relatives, without fear.”* (Interview 4, Pos. 211–214).

#### 3.1.2 Segregation of dying and death

The interviewed veterinarians also discuss the institutional relocation of old, sick, and dying individuals in terms of the described segregation from death and dying. In one interview, it is stated: “*In human medicine, it is indeed the case that many people die in institutions, in hospitals or nursing homes, and only a few of us go to bed and do not wake up, completely unspectacularly, without a huge story of suffering beforehand or without any signs being visible […]”* (Interview 5, Pos. 89). Another veterinarian notes that the handling of dying strongly depends on the individual person and their surroundings; however, there is a tendency for people to increasingly have less direct confrontation with the dying of others: “*So, one cannot generalize this for everyone; it always depends on the individual, but yes, I do believe that death is something we are no longer confronted with in our everyday lives, or that it often takes place in nursing homes and hospitals, and much of it is not really seen”* (Interview 2, Pos. 187). The following quote serves as a succinct summary of the observation regarding the “relocation” of dying and death: “*In society, […] aging, illness, and death no longer exist per se. Because you are in the hospital, in a home, in hospice. And the last thing people want to see are the grandparents who are wasting away; they don't go to see them at all. And to be honest, I believe that death does not hold much significance in our society.”* (Interview 1, Pos. 275).

#### 3.1.3 Fear and uncertainty regarding death

The respondents also address a central reason for the taboo and suppression surrounding the topic: the veterinarians perceive a significant level of fear and uncertainty in contemporary society—also among their clients—when it comes to dealing with “dying” and “death.” One interviewee describes the following: “*Through accompanying a sick friend, I've gained a lot of experience and, above all, I've realized how uncertain people are. They often choose to stay away, and that is so painful, right? This hesitation to remain present out of fear of not being able to handle it or of saying or doing something wrong is truly one of the bitterest experiences. Because at that moment, both people and animals need companionship – companionship from those they know and trust, you know?”* (Interview 6, Pos. 15).

A veterinarian with extensive professional experience describes the following case, noting that this fear and uncertainty often stem from the fact that we have little direct experience with dying and death in our immediate social circles: “*Back in my day, when grandpa passed away, you would go over and see him right after he had died. That doesn't really happen anymore. Many people don't even know what happens when someone dies. And that's what they fear when they are alone with their pet. I believe this is a major reason. It's not always just about whether the animal is suffering*.” (Interview 14, Pos. 108).

### 3.2 Dying and death of companion animals

When veterinarians describe their observations regarding how the dying of companion animals is perceived and the consequences that arise, they address various aspects. They particularly emphasize that accompanying a dying companion animal has the potential to bring death out of the “taboo zone” and to provide valuable first hand experiences in dealing with the dying of others as well as with one's own mortality.

#### 3.2.1 De-tabooing and visibility of death through experiences with companion animals

As previously shown, the respondents describe death as a societal taboo. However, they also emphasize that experiences with a dying or deceased companion animal can counteract this taboo. A veterinarian states: “*Being present for the dying or loss of a pet naturally brings death out of the taboo zone. If I don't have pets and have no connection to death at all, then this wall of the taboo zone can be particularly high and dense.”* (Interview 6, Pos. 195).

This experience is not limited to adults; rather, accompanying a dying companion animal can have a particularly positive impact on children in this context, as “*this taboo is then removed”* (Interview 16, Pos. 140). One veterinarian shares the following: “*I believe that euthanizing a pet or accompanying a pet through death, when we experience it ourselves, makes it less taboo. […] And yes, I am sure that it contributes to breaking down the taboo, especially for children. Children can also bury the pet in the garden, participating in the whole process after its death, and I think it's a positive experience for them to witness.”* (Interview 19, Pos. 155).

#### 3.2.2 The dying of companion animals as a “training ground” for dealing with death

The respondents view accompanying the dying of companion animals not only as a counterpoint to the taboo surrounding this topic but also as a kind of “training ground” that fosters a reflective approach to death—whether concerning others or oneself. One veterinarian emphasizes: “*I believe that, especially for children, this is a good training ground. I also find that children often have fewer problems with death. They seem much closer to that other dimension. But it's also true for adults. I think it's like a training ground, which might sound silly, but for some people, pets are almost closer than relatives or other people.”* (Interview 8, Pos. 139).

One of the interviewed veterinarians emphasizes that children can learn to cope with the finiteness of life early on through the loss of a companion animal. She describes how this experience can contribute to the development of important emotional skills: “*In families, when children learn that life ends and that there's nothing they can do about it, it's crucial for them to accept the unchangeable and to endure the frustration that comes with something happening that they really don't want. This is immensely helpful, right? It teaches people that here is something unchangeable, something that happens, and it's not a taboo zone.”* (Interview 6, Pos. 195).

One interviewee describes her experience with her own child, highlighting how the dying and death of their hamster and cat were important primary experiences: “*I honestly experienced this myself. I thought, ‘Great, now she has experienced this.' When my parents pass away, she will have already gone through it with the pet. It's like a step, an intermediate step. First, it was a hamster we had; now it's the cat. I don't know what will come next, and at some point, if everything goes normally, my parents will pass away, her grandparents.”* (Interview 16, Pos. 140).

A veterinarian points out that accompanying a companion animal until its end can also provide a hopeful perspective on one's own death: “*It also allows something to change within the person. The pet facilitates this. The person becomes more mature through this experience. […] Those who have gone through this now have hope for their own death. They know that in 30 years, if all goes well, they will die, right?”* (Interview 9, Pos. 169, 173).

The quotes presented thus far illustrate that accompanying a dying companion animal is viewed by the interviewees as a potentially positive primary experience. At the same time, the veterinarians discuss how this context generates questions and discussions with companion animal owners, particularly regarding the inclusion of children. They describe how parents often approach them with the question of whether children should be present during the euthanasia of the companion animal. With respect to this, a veterinarian shares: “*I do believe that it has an impact on young people when they see how it all unfolds. The question is whether they want to be there. I often get asked, ‘I have a daughter; do you think it makes sense for her to witness this? When should we involve her?' My opinion is that death is part of life, and we tend to push it too far away. I find it reasonable to observe it. You don't have to go through it in epic detail, but you should at least understand what's happening and what the outcome will be—that in the end, there will be a dead dog or cat, and that's a final story. I do think it has an influence.”* (Interview 12, Pos. 181).

Another veterinarian argues similarly, noting that dying and death in the case of euthanasia can be seen as a form of release: “*I believe that, in principle, it is a valuable experience, and I also think it's not something that we need to protect children from. […] Especially in the case of… well, perhaps the death of a pet can show people that it doesn't have to be such a terrible thing. It can also in the case of a prolonged illness, be a release. So, it's not only negatively connoted.”* (Interview 2, Pos. 187).

In particular, the study's results indicate that experiencing the death of a companion animal can help reduce fears about death, according to the respondents. One veterinarian describes that “*the fear of death, which most people have, diminishes. In fact, very few people who have experienced this [the death of animals] still fear death. They have no fear of death or dying, not even for themselves. They gain a completely different worldview. […] This means that all the fear surrounding aging, death, and the problems that come with it dissipates.”* (Interview 4, Pos. 203). Another veterinarian emphasizes that, particularly among companion animal owners who have already experienced the loss of an animal, the fear of dealing with dying and death significantly decreases: “*We also have pet owners with multiple animals, who we helped with their first pet, and they had a panicked fear of the pet's death. But with the second pet, they navigated the situation so confidently and managed it so well that you could really see they were proud of themselves.”* (Interview 13, Pos. 149).

#### 3.2.3 Negative experiences and their impact on coping with the death of companion animals

The interviewed veterinarians emphasize not only the positive potentials associated with accompanying a companion animal until its end—such as regarding one's own approach to dying and death or the loss of loved ones—but also highlight contrasting dynamics. On one hand, individuals who tend to suppress aging, illness, suffering, and dying in others may also be inclined to ignore these experiences in their companion animals. One veterinarian notes that some companion animal owners struggle when their animals age and exhibit age-related frailties: “*I do encounter some pet owners who want to avoid all of this, because there are indeed pet owners who cannot see what they always refer to as suffering. I'm not always sure what they don't want to see, because a dog… of course, an old dog is often just as frail as an old person. They're not as fit anymore. And some pet owners truly cannot bear to witness that.”* (Interview 11, Pos. 37).

On the other hand, negative experiences of companion animal owners related to illness, aging, or previous losses (whether human or animal) can significantly burden and complicate the farewell to their own companion animals. One veterinarian recounts a situation involving a companion animal owner who developed a considerable aversion to confronting death due to the traumatic experiences surrounding her husband's death from cancer. These negative experiences also affected her ability to accompany her cat through its illness. The veterinarian explains: “*There are indeed pet owners who have had different experiences. I knew that her husband had died of cancer. […] I believe that accompanying him until his death was not pleasant for her. She was quite traumatized by the whole thing. […] And her cat, yes, her cat had a lymphoma […] she said no. At that time, she stated that she couldn't bear to see this again.”* (Interview 11, Pos. 13). These experiences led the owner to refuse potential treatment, ultimately shortening her cat's lifespan.

## 4 Discussion

This article focused on two overarching research questions: First, how do veterinarians working in palliative care and end-of-life support perceive society's engagement with dying and death in general, and, second, what significance do they attribute to the dying and death of companion animals in this context. Regarding the first question, the veterinarians appear as advocates of the taboo or displacement thesis: The respondents diagnose a deeply rooted form of repression or taboo surrounding dying and death in contemporary Western society. Terms like “death is somewhat a taboo,” “it is being anonymized,” “treated very negligently,” and “simply not discussed” support the phenomenon described by Foucault in the mid-20th century regarding the increasing disappearance of public rituals surrounding death (1). Medical professionals in human medicine often share the view that death is a taboo subject in society, one people prefer not to discuss and would rather repress (26, 27).

The societal taboo surrounding death (8–10), according to the interviewed veterinarians, manifests particularly in the institutional segregation of old, sick, and dying people. Moreover, it is expressed, according to the veterinarians, in forms of uncertainty or even fear in everyday interactions with both the topic of death and with dying individuals themselves. Notably, the respondents often describe their observations and perceptions through cultural comparisons. They refer to cultures where death and dying are, in their view, still a communal experience, emphasizing the positive collective aspect. Social science shows that perceptions of death vary significantly across cultures, shaping societal approaches to the dying process (28). Sociologist Thieme, for example, emphasizes that how people materially and mentally deal with death is not random or inherently natural; instead, it is guided by cultural norms and conventions. As a result, individuals' understanding of death and their reactions to it are influenced by their cultural backgrounds (29). While in other cultures mourning is ritualized in public spaces, for example through death dances at funerals (30), a privatization of mourning and grief management has established itself in Western countries. In a study conducted in Germany and Switzerland, nearly a quarter of 355 participants indicated that mourning should take place privately rather than in public, and half felt more comfortable keeping their grief to themselves (31).

The interviewed veterinarians describe significant cultural differences in attitudes toward death, highlighting pervasive taboos associated with dying, death, and mourning in certain Western contexts. Undoubtedly, this raises the question of which specific countries or cultural contexts the respondents—who come from three Central European countries—are referring to and, more fundamentally, whether such generalizing statements about an increasingly heterogeneous contemporary society can be considered valid at all.

Further, the privatization of mourning and grief management not only exacerbates the emotional burden faced by grieving individuals but also complicates the end-of-life process for veterinary professionals guiding them. In relation to this, it is important to acknowledge that statements made by veterinarians may sometimes lead to broad generalizations about societal attitudes. Often, these claims are rooted in individual experiences, which can result in conclusions that do not fully represent the diversity of perspectives on death and dying. For instance, assertions about limited direct experiences with mortality may be based on anecdotal evidence, potentially reinforcing uncritical views of how society perceives these complex topics. Despite these concerns, veterinarians advocate for a more open, ritualized, and shame-free approach to death and dying. They believe that addressing these taboos can foster healthier discussions about grief, allowing for shared experiences and communal support. By normalizing conversations around these topics, veterinarians aim to create an environment where pet owners feel comfortable expressing their emotions, seeking support, and engaging in meaningful rituals that honor their pets' lives. Such a shift toward openness could improve the quality of end-of-life care by encouraging veterinarians to engage more empathetically with clients, tailoring their support to the unique needs of each grieving individual. Ultimately, fostering a more inclusive dialogue about death may not only alleviate the emotional burdens of grieving but also enhance the overall experience of farewell, transforming it into a more compassionate and supportive journey for all involved.

The second overarching research question also reveals a clear trend in the responses: according to the interviewees, accompanying a dying companion animal offers opportunities to counteract the societal repression and taboo surrounding death and to give dying a new visibility. The veterinarians describe the conscious care of companion animals in their final stages as a “training ground” that encourages a more reflective and open approach to death, not only concerning the death of an animal but also in relation to the dying of fellow humans. The term “training” should not be misunderstood; it does not imply the instrumentalization of animals, as Schopenhauer criticized Kantian ethics for suggesting that one should feel compassion for animals “merely for practice” (32). Instead, the veterinarians view the feelings of grief and loss experienced with a companion animal's death as authentic, crisis-like experiences, which unintentionally lead to a more reflective engagement with death. By discussing the loss of their dog or cat and the associated emotions, families can foster a culture of openness that positively influences their general approach to dying. Thus, the death of a companion animal could serve as a catalyst for destigmatizing death and the accompanying grief.

In some narratives from the respondents, accompanying a dying animal is even described as potentially beneficial for developing a more reflective attitude toward one's own mortality. Viewed in the context of the history of ideas, these responses can be seen as aligned with the tradition of Stoic philosophy. Stoicism considers the constant awareness of life's finitude not only essential for a good, virtuous life, but also describes the conscious engagement with one's mortality as something that *can* and *should* be trained (33). The loss of a companion animal can help foster a more sensitive and conscious approach to death and dying, both for humans and animals. However, the interviewed veterinarians also suggest that there could be the opposite effect: negative experiences with the death and dying process of close individuals can influence companion animal owners' behavior and decisions regarding their companion animal's end of life, sometimes in distressing ways. For instance, if a dog owner has recently lost her husband after a long period of caregiving, she may feel emotionally overwhelmed when it comes to providing palliative care for her animal, even if the medical prognosis indicates a longer phase of good quality of life. Therefore, experiences related to illness, caregiving, aging, and the end of life for loved ones can impact decisions, desires, and fears concerning one's own companion animal. In relation to the aspect of fear, it is important to note that the fear of death is a multifaceted issue that extends beyond the mere fear of dying. Respondents in our study indicated that experiencing the death of a companion animal can alleviate some fears related to mortality; however, it is essential to recognize the broader dimensions of this fear. Individuals often grapple with anxieties surrounding not only their own death but also the pain associated with dying, the suffering of loved ones, and the emotional aftermath of loss. Moreover, the impact of traumatic human loss on how individuals respond to the death of a pet cannot be overlooked. For some, the death of an animal may reinforce avoidance behaviors and exacerbate existing fears. Those who have experienced significant human loss may find themselves more apprehensive about facing the reality of animal death, potentially leading to a reluctance to engage with the dying process. This avoidance can complicate their emotional responses and hinder healthy grieving. Additionally, witnessing euthanasia can introduce new anxieties. The experience of making the decision to end a pet's life can evoke feelings of guilt and helplessness, which may trigger unresolved grief from past losses. Such experiences can create a complex emotional landscape, where individuals confront not only their own fears of mortality but also the pain of making difficult decisions for their beloved companions. As one veterinarian noted, the transformation in worldview that comes from facing the death of a pet allows individuals to confront their own mortality with greater confidence. Yet, this does not negate the complexity of their fears. Understanding the nuanced relationship between animal loss, traumatic human loss, and the fear of death can provide deeper insights into how pet ownership influences attitudes toward mortality. This suggests a need for further exploration into the psychological and emotional processes at play. Future research should systematically examine these connections and potential interactions between human medicine and veterinary medicine.

Current research indicates that veterinarians specializing in palliative care and hospice are expected to engage in roles that extend beyond traditional medical duties (21, 34), providing essential emotional support and guidance to companion animal owners during the end-of-life process. Further, veterinarians often act as “counselors” or “social workers,” addressing the problems, wishes, fears, and personal histories of companion animal owners. In this context, they can be viewed as “experts on death,” as described by Walter (7), to whom fundamental questions about the end of life are directed. Furthermore, it can be anticipated that in discussions surrounding death, these veterinarians may also assume a specific role, facilitating conversations and helping owners navigate their grief and decision-making. A vivid illustration of this aspect is provided by a veterinarian who shares that parents ask her whether their children should be present during their animal's euthanasia—essentially, whether it is appropriate for a child to witness the dying process. In such cases, the veterinarian is seen not only as a medical expert but also as someone with specialized experiential knowledge and a unique perspective on death and dying that is not readily accessible to the general public. This situation draws a loose parallel to earlier professional roles: Walter (7) and Elias (35) describe how, in pre-modern societies, the priesthood was viewed as a key societal authority in matters of death and dying, holding a sort of “monopoly of knowledge” in these areas. In modern society, this previously religious interpretative privilege is partially supplemented or replaced by medical personnel, which aligns with Walter's (7) argument that suggests a shift in authority from “priest” to “doctor” in the transition from a “traditional” to a “modern” death. The present study adds that veterinarians also emerge as relevant actors in these discussions, being granted a comparable interpretative and guiding framework in specific contexts.

Veterinarians play a multifaceted role in the end-of-life care of companion animals, extending beyond their medical expertise (19–21, 23, 34). Based on recent investigations and the current study, it can be argued that veterinarians—especially in this field—can be recognized as “experts on death,” providing essential emotional support to pet owners during this challenging time. Their involvement often occurs in intimate settings marked by profound grief, where they not only address the physical aspects of euthanasia but also guide families through the emotional complexities of saying goodbye. This dual role underscores the importance of veterinarians as compassionate allies in the grieving process, highlighting their unique position to support both the animal and the owner in navigating the difficult journey of loss. This result highlights that their daily work requires skills that extend far beyond pure medical expertise. However, current literature suggests that veterinarians feel inadequately prepared to handle issues related to death, dying, and the grief of owners, indicating room for improvement (36). This underscores the need to systematically incorporate topics such as death, grief, and end-of-life care into veterinary education and continuing education programs. Only by doing so veterinarians can be appropriately supported in their roles as medical experts at the end of life, preparing them for the diverse demands of their profession. Paraphrasing Stoic philosophy: Just as individuals can and should practice dealing with their own mortality, the same applies to supporting owners and their dying animals.

While this study offers valuable and innovative insights into the dynamics of dying and death from a thanatosociological perspective, it is important to acknowledge several limitations. Firstly, the sample size and selection may not fully represent the broader population of veterinarians specializing in palliative care and hospice services. The respondents' experiences and perspectives could vary significantly based on geographical location, cultural background, or specific professional training, which may limit the generalizability of the findings. Secondly, the reliance on qualitative interviews may introduce subjective biases, both from the participants and the researchers. While qualitative data provide rich insights, they may not capture the full complexity of the issues surrounding death, dying, and grief in veterinary practice. Additionally, the study primarily focuses on the experiences of veterinarians and does not include the perspectives of owners or other stakeholders in the end-of-life process. The voices of owners could provide essential context and depth to the understanding of the emotional and psychological impacts of losing a companion animal. Moreover, the evolving nature of veterinary education and training may mean that some of the findings become outdated as new curricula and approaches are developed. Future research should aim to incorporate a more diverse range of participants and perspectives, as well as explore longitudinal changes in attitudes and practices related to end-of-life care in veterinary medicine.

## 5 Conclusions

In conclusion, this article highlights the complex interplay between societal perceptions of death and the experiences of veterinarians specializing in palliative care and end-of-life support for companion animals. The findings reveal a significant taboo surrounding death in contemporary Western society, as articulated by the veterinarians, who advocate for a more open and communal approach to dying. Their reflections on cultural differences emphasize the need for societal change in how we engage with death, drawing attention to the emotional burdens that a privatized approach can impose.

Furthermore, the veterinarians' insights suggest that the process of accompanying a dying companion animal not only provides a space for grief but also serves as a catalyst for broader reflections on mortality, both for owners and society at large. The dual role of veterinarians—as medical experts and emotional supporters—underscores the importance of integrating discussions about death and grief into veterinary education and professional development. By fostering skills that extend beyond medical knowledge, veterinarians can better support owners through the challenging end-of-life journey.

## Data Availability

The original contributions presented in the study are included in the article/Supplementary material, further inquiries can be directed to the corresponding author.
