# Supplementary material for: Beyond the taboo: a thanatosociological investigation of companion animal loss based on an interview study with small animal veterinarians
Source: Front Vet Sci. 2026 Jan 30;13:1719122. doi: 10.3389/fvets.2026.1719122 (PMC12902952; doi:10.3389/fvets.2026.1719122)
Supplement: Supplementary file 1 [file Supplementary_file_1.docx]

Supplementary Material 1

# Overview of selected characteristics of interview participants

| **Interview (Int.)** | **Country** | **Gender** | **Self-employed or employed** | **Professional experience in years** | **Duration of the interview** | **Interviewer** |
| --- | --- | --- | --- | --- | --- | --- |
| INT_1 | Austria | Female | Self-employed | 25 | 01:29:12 | S.S. |
| INT_2 | Germany | Female | Self-employed | 8 | 01:20:52 | S.S. |
| INT_3 | Germany | Female | Self-employed | 30 | 01:18:46 | S.S. |
| INT_4 | Germany | Female | Self-employed | 23 | 01:06:32 | S.S. |
| INT_5 | Austria | Female | Self-employed | 15 | 01:40:10 | S.S. |
| INT_6 | Germany | Female | Employed | 32 | 01:27:13 | S.S. |
| INT_7 | Germany | Male | Self-employed | 30 | 01:13:34 | S.S. |
| INT_8 | Switzerland | Female | Self-employed | 35 | 0:47.57 | C.D. |
| INT_9 | Switzerland | Female | Self-employed | 28 | 01:26:38 | S.S. |
| INT_10 | Austria | Female | Self-employed | 19 | 00:54:29 | C.D. |
| INT_11 | Austria | Female | Self-employed | 23 | 01:18:55 | S.S. |
| INT_12 | Germany | Female | Self-employed | 12 | 00:50:04 | S.S. |
| INT_13 | Austria | Female | Self-employed | 12 | 01:28:39 | S.S. |
| INT_14 | Germany | Female | Self-employed | 35 | 01:14:04 | S.S. |
| INT_15 | Switzerland | Female | Self-employed | 16 | 01:18:02 | S.S. |
| INT_16 | Switzerland | Female | Self-employed | 15 | 01:13:54 | S.S. |
| INT_17 | Switzerland | Female | Self-employed | 20 | 00:53:28 | S.S. |
| INT_18 | Germany | Male | Self-employed | 22 | 00:53:15 | S.S. |
| INT_19 | Switzerland | Female | Self-employed | 17 | 01:07:51 | S.S. |
| INT_20 | Germany | Female | Self-employed | 28 | 01:15:22 | S.S. |
